# ZnO Nanostructures for Drug Delivery and Theranostic Applications

**DOI:** 10.3390/nano8040268

**Published:** 2018-04-23

**Authors:** Marina Martínez-Carmona, Yurii Gun’ko, María Vallet-Regí

**Affiliations:** 1 School of Chemistry and CRANN, Trinity College, The University of Dublin, Dublin 2, Ireland; martim10@tcd.ie (M.M.-C.); igounko@tcd.ie (Y.G.); 2 Department Chemistry in Pharmaceutical Sciences, School of Pharmacy , Universidad Complutense de Madrid, Instituto de Investigación Sanitaria Hospital 12 de Octubre i+12, 28040 Madrid, Spain; 3 Centro de Investigación Biomédica en Red de Bioingeniería, Biomateriales y Nanomedicina (CIBER-BBN), Avenida Monforte de Lemos, 3-5, 28029 Madrid, Spain

**Keywords:** ZnO nanoparticles, Quantum dots, theranostic, drug delivery, anti-tumour, diabetes treatment, anti-inflammation, antibacterial, antifungal, wound healing

## Abstract

In the last two decades, zinc oxide (ZnO) semiconductor Quantum dots (QDs) have been shown to have fantastic luminescent properties, which together with their low-cost, low-toxicity and biocompatibility have turned these nanomaterials into one of the main candidates for bio-imaging. The discovery of other desirable traits such as their ability to produce destructive reactive oxygen species (ROS), high catalytic efficiency, strong adsorption capability and high isoelectric point, also make them promising nanomaterials for therapeutic and diagnostic functions. Herein, we review the recent progress on the use of ZnO based nanoplatforms in drug delivery and theranostic in several diseases such as bacterial infection and cancer.

## 1. Introduction

For many years, the use of organic dye molecules has allowed us to detect and monitor various kinds of substances, including drugs, amino acids, nucleotides or materials, both in and outside of cells. They have also been used to study the process of life chemistry (enzymatic synthesis, immune response, etc.) or to identify some diseases. However, its use in bio-imaging has been drastically reduced since the appearance of the quantum dots (QDs) [1]. In general, QDs are more stable to photochemical degradation, have wide excitation wavelength ranges and narrow and symmetric emission spectra and can exhibit different colours depending on the size of the particle [2] (the so-called quantum size effect) [3].

Among the typical QDs, i.e., CdSe, CdTe, CuO, TiO_2_, etc., ZnO are without any doubt one of the best choices since they are excellent semiconductors, with luminescent properties [4,5] almost as good as those of the Cd QDs ones but presenting the advantage of being biodegradable and nontoxic [6]. In fact, although its effect at the nanometre level has not yet been established, ZnO in bulk has already been considered as safe and approved by the US Food and Drug Administration [7].

ZnO is an n-type semiconductor with an outsized exciton-binding energy (60 meV), a wide band gap of 3.37 eV at room temperature, a Bohr exciton radius of ~2.34 nm and a high dielectric constant. Irradiatation of ZnO with UV light favours the promotion of an electron (e^−^) to the conduction band and therefore producing a hole (h^+^) in the valence band, namely the electron/hole pair. Apart from this typical UV range excitonic emission, the photoluminescence spectrum of ZnO nanocrystals also displays a broad visible emission, more suitable for biological imaging. This extended emission has been ascribed to point defects such as O and Zn vacancies or interstitials and related to surface oxygen-containing moieties, such as OH groups [8]. 

Moreover, the luminescence of ZnO nanocrystals can be improved or modulated by doping the structure with other ions [9,10].

In addition, ZnO QDs also present properties such as the ability to produce ROS, a strong adsorption capability and an easily tuneable surface that play a crucial role in their use for biomedical applications.

When ZnO crystals are under UV irradiation in aqueous suspension, these electron/hole pairs will produce several photochemical reactions generating ROS, making them good candidates for photodynamic therapy [11]. Usually, when ZnO QDs are excited, the valence band holes present on the surface, abstract electrons from water and/or hydroxyl ions, giving place to hydroxyl radicals (OH^•^). At the same time, the superoxide anion O_2_^-^ is produced due to the reduction of oxygen [12]. Apart from the high production of ROS after UV irradiation, ZnO QDs themselves can also generate small amounts of ROS due to the pro-inflammatory response of the cell against nanoparticles (NPs) [13] and to the characteristic surface property of ZnO QDs [14,15]. Normally, UV light is required to produce these electron/hole pairs, however, for ZnO particles whose size is on the nanometre scale, electrons can also reach the conduction band without the help of UV excitation [16], probably because of the presence of crystal defects due to their nano-size. Fortunately, this phenomenon is of little importance outside the cells, where the concentration of ROS is small, but once internalized higher levels of ROS resulting in cell death. Some studies reveal that ROS production is significantly higher in tumour cells than in normal ones after being treated with ZnO QDs [17]. It has been reported that various signalling molecules and ROS are generally more abundant in cells such as tumour cells, due to a rapid metabolic rate, and high degree of growth and multiplication than in normal cells [18].

ZnO QDs have also be produce a variety of different nano-architectures, including nanospheres, nanorods, nanotubes, nanorings, nanobelts, nanoflowers, etc. [19,20,21,22,23].

ZnO QDs have a versatile surface chemistry that can easily be modified to prevent aggregation, improve colloidal stability [24] or to obtain new properties as drug delivery systems (DDS) [25,26].

The use of DDS in nanomedicine has important advantages compared with traditional drugs: (i) increasing solubility of drugs that cannot be taken up by cells and increasing therefore their bioavailability [27,28]; (ii) avoiding the degradation of some drugs that are unstable at physiological or gastrointestinal pH [29,30]; and (iii) reducing the toxicity and side effects of drugs by using target molecules that increase the selectivity of the treatment [31,32].

Considering their ability to produce ROS, capacity to act as drug delivery systems and their luminescence properties, we can talk about theranostic nanoplatforms where ZnO QDs not only perform the role of image agents but also of treatment [33,34].

**Figure 1:**
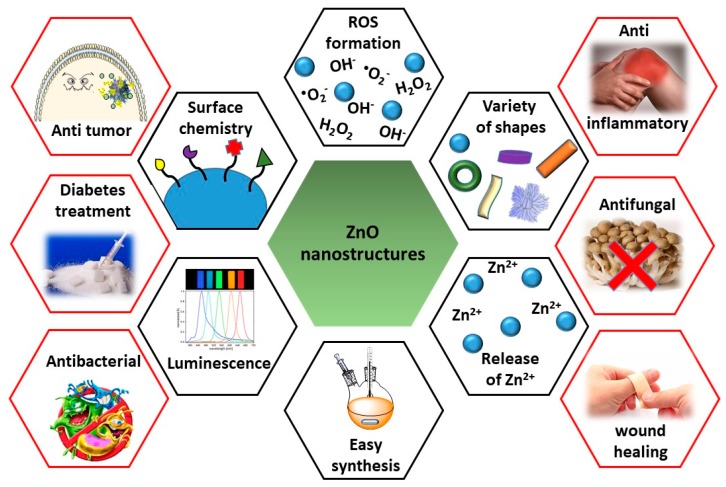
Diagram summarizing the main characteristics of ZnO nanostructures (black hexagons) and their principal applications in biomedicine (red hexagons).

Herein, in this review, we will summarize the recent progress on the use of ZnO QDs for drug delivery and theranostic imaging in different pathologies (Figure 1). Approaches to the preparation and chemical functionalization of ZnO nanostructures for biological applications are very well documented and this area was a subject of several recent reviews [7,35,36,37,38,39], therefore we are not going to consider these aspects in this manuscript.

## 2. ZnO Nanoplatforms for Theranostic in Cancer

In view of the number of publications over the last years, there is no doubt that cancer is the main objective in terms of the use of ZnO based materials for the treatment of diseases. ZnO QDs slowly dissolve in physiological pH [40] producing small changes in extracellular zinc concentrations that cause very little cytotoxicity. However, NPs preferentially internalized in tumour cells as consequence of the enhanced permeability and retention (EPR) effect. Once inside and because of electrostatic interactions, ZnO QDs present certain cytotoxicity by themselves based on a higher intracellular release of dissolved zinc ions due to the acidification of the media, followed by increased ROS induction. This situation results in the loss of protein activity balance mediated by zinc as well as in an oxidative stress environment that finally produce cell death [41].

In addition to the synthetic versatility of these materials, we find that ZnO can act as a core, as a shell around other types of particles or provide an added value to more complex systems. All the systems are summarized and referenced in Table 1.

### 2.1. ZnO Core Nanosystems

**Figure 2:**
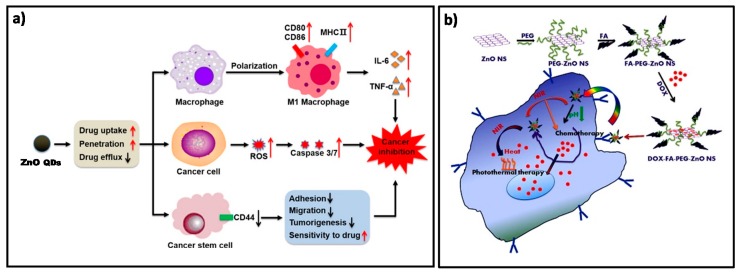
(**a**) Scheme of the multiple proposed effects of ZnO QDs as a multi-functional antitumour treatment. Reproduced with permission from [44]. American Chemical Society, 2017; (**b**) Scheme of the combined mechanism of action of DOX-FA-ZnO NS for breast carcinoma therapy. Reproduced with permission from [48]. Elsevier, 2017.

Regarding systems that use ZnO as core for the treatment of cancer, in 2016, Vaidya et al. adsorbed doxorrubicin (DOX) onto the surface of ZnO QDs (ZD QDs) and studied their anticancer activity in MCF-7 cells compared to that presented by free DOX, ZnO QDs, and a mixture of the latter two. It was observed that the combined addition of ZnO QDs and DOX presented higher antitumour capacity than any of its components separately but lower than the effect of ZD QDs, maybe because of a better targeting and a higher retention of the DOX loaded QDs in the tumour cells [42]. At the same time, Liang et al. performed a similar study with MCF-7R and MCF-7S cells. In this case, they explained the release of the drug due to the degradation of ZnO in response to pH after internalization of the QDs into the endo/lysosomes. They also performed a real-time tracking of the drug release. Although the two components separately exhibited fluorescence, their intensity was quenched after ZD QDs formation. However, after the degradation of ZnO and the consequent release of the DOX, the intensity of the fluorescence increased again [43]. In 2017, Zhu et al. went one step further and proposed ZnO QDs as a multifunctional platform for cancer treatment (Figure 2a). They studied the synergistic anticancer activity due to the ROS generation of ZnO QDs and DOX in several cell lines but also studied their effect in macrophages or in tumour (stem-like) cells. In stem cells, it was observed that ZnO QDs affected the expression of CD44, leading to a marked decrease in migration, accumulation of mutations and cell adhesion, but increasing sensitivity to antitumour treatment. In macrophages, a polarization towards the phenotype M1 was observed, increasing the antitumour effectiveness and immune response of DOX [44].

Bahadur et al. demonstrated that the application of ultrasound irradiation in ZD QDs can be used for on-demand pulsatile release of DOX molecules [45]. To increase the selectivity and luminescence of the nanocarrier, Zhu et al. designed Mg ZnD QDs functionalized with Folic Acid (FA) and studied their toxicity in HeLa cells [46]. Pathak et al. also used FA as targeting agent to synthesize a new ZnO hollow-nanocarrier containing paclitaxel as model drug. Initially, they suspended carbon spheres in zinc acetate solution and added ammonia to form a zinc hydroxide layer onto the surface of the carbon spheres. After that, the carbon was removed by pyrolysis, giving rise to the hollow ZnO spheres. Then, the NPs were loaded with paclitaxel and functionalized with FA. The effectiveness of the nanosystem was successful both in vitro by producing cytotoxicity with breast cancer cells and in vivo by reducing MDA-MB-231 xenograft tumours in nude mice [47]. FA modified zinc oxide nanosheets (Ns) were also proposed by Kannan et al. as a chemo-photothermal device for breast cancer therapy. The experiments showed that the combination of both therapies (chemotherapy and photothermal therapy) resulted in higher percentages of cell death than either of them separately. In addition, in vitro and in vivo experiments showed no adverse effect or toxicity on blood stream. In Figure 2b, a scheme of the combined mechanism of action of these nanosheets is presented [48]. As explained in the Introduction, the irradiation of ZnO QDs with UV light increases the production of ROS, enhancing the antitumour capacity of the QDs. Several groups have studied this effect in tumour cells with pure particles [12], particles doped with other ions [33], in combination with different antitumour agents [49,50] or using an aptamer as targeting agent [51].

### 2.2. ZnO Core Nanocomposites

**Figure 3:**
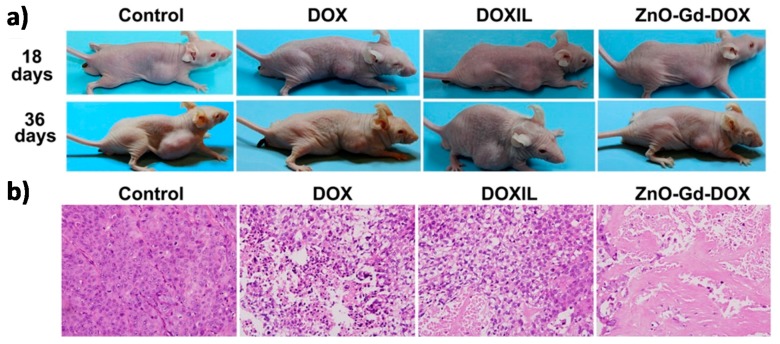
(**a**) Images of BxPC-3 tumour-bearing nude mice after 18 and 36 days under different treatments; (**b**) H&E staining of tumour slices after 36 days of treatments by different agents. Reproduced with permission from [34]. American Chemical Society, 2016.

Several nanocomposites based on ZnO QDs in combination with other materials to obtain new nanocomposites with synergist theranostic effects have also been reported. For example, H. Möhwald et al. synthesized ZnO QDs with a polymeric shell, coordinated with Gd^3+^ ions and adsorbed DOX to create a versatile ZnO-Gd-DOX nanodevice. It was a bifunctional probe for both in vitro fluorescent and in vivo animal imaging, due to the strong red emission of ZnO-Gd-DOX in the range of 600–800 nm and magnetic resonance imaging (MRI) contrast due to Gd^3+^ ions, which were immobilized onto the ZnO surface through proper coordination with carboxyl groups of the polymer. This rendered an outstanding relaxivity for MRI. Most importantly, these nanomaterials also demonstrated a very promising antitumour activity. BxPC-3 tumour-bearing nude mice were injected with different agents to study chemotherapy efficacy. As shown in Figure 3a, the tumour in the control continued growing, while those treated with DOX, Doxil or ZnO-Gd-DOX QDs remained more or less the same. H&E (hematoxylin and eosin) staining of tumour slices (Figure 3b) showed that the cells in the control retained their normal membrane and nuclear structures, and the cells treated with DOX or Doxil were damaged partly, while almost all cells were severely destroyed after ZnO-Gd-DOX treatment. This result confirmed that ZnO-Gd-DOX QDs performed better than the other agents. Finally, they also observed that ZnO-Gd-DOX QDs had no detectable toxic side effects to mice, and the whole QDs could be biodegraded and excreted from the mice body [34].

Qu et al. combined the advantages of mesoporous silica nanoreactors, DOX, FA, and ZnO QDs to develop a drug carrier effectively protected from non-specific degradation (ZnO-DOX@F-mSiO_2_-FA). They demonstrated that the mesoporous silica shell protected the ZnO-DOX device from non-specific protein degradation, while retained its sensitivity to pH-responsiveness. To perform the experiments, HeLa and HEK 293T cells, which are, respectively, positive and negative for folate receptor, were selected as model cells. After the treatment with ZnO-DOX@mSiO_2_-FA, a clear increase in positive annexin V-FITC HeLa cells was observed when compared with control ones. The results also showed an effective targeting due to the presence of the folic acid as ZnO-DOX@mSiO_2_-FA presented selective toxic capacity toward HeLa cells [52]. Cauda et al. designed new lipid-coated ZnO nanocrystals (NCs) to achieve a better stability in biological samples. Their results showed that lipid-coated ZnO NCs presented stable colloidal dispersions in cell culture medium and simulated human plasma for 25 days. However, after being suspended, the pristine and amine-functionalized NCs quickly aggregated, remaining stable for less than an hour. Even though internalization of lipid-shielded ZnO NCs in HeLa cells was higher compared to the other samples, its toxicity was lower, showing a lower toxicity/particle ratio [53].

### 2.3. ZnO Coated Nanodevices

**Figure 4:**
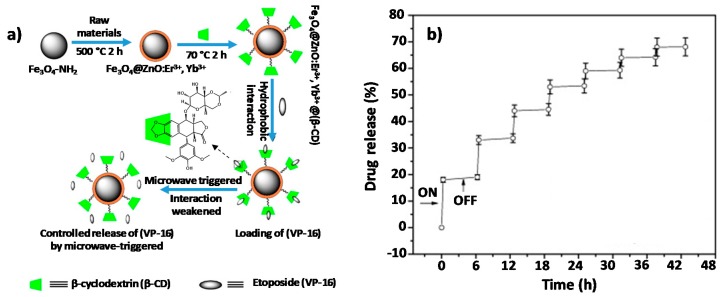
(**a**) Synthesis scheme and mechanism of action of Fe_3_O_4_@ZnO:Er^3+^,Yb^3+^@β-CD nano-composites; (**b**) Graph of VP-16 release from the Fe_3_O_4_@ZnO:Er^3+^,Yb^3+^@(β-CD)–(VP-16) depending on the number of microwave cycles applied. Reproduced with permission from [55]. Elsevier, 2015.

Due to its high biocompatibility, in recent years ZnO has been used as a coating for other types of QDs that present more toxicity. In comparison with the pegylation that also provides stability and biocompatibility, the use of ZnO as a coating provides added values such as luminescent properties or ROS production, among others, that cannot be achieved by PEG functionalization. For instance, H. Danafar et al. recently reported a new system, TiO_2_@ZnO NPs, where ZnO is used to coat mesoporous TiO_2_ QDs. They loaded the mesopores with curcumin (Cur) and studied their pH-dependent in vitro anticancer effect against human epithelial colorectal adenocarcinoma cells. The cytotoxic capacity was also compared with a similar system that contained graphene oxide (GO), (which has probe to be quite efficient in the treatment of cancer of colon) [62,63] as final layer, TiO_2_@ZnO–GO. Opposite to what was expected, the presence of GO did not increase toxicity. Proof of this is that TiO_2_@ZnO showed higher killing capability against Caco-2 cancer cell than the ones that contained GO. Therefore, there was reduced toxicity of ZnO nanoparticles [26]. Authors suggested that effect could be because of the presence of ZnO nanoprecipitation on the tumour cells [64,65]. In 2015, Wang et al. reported two different systems based on iron oxide QDs coated with ZnO and sensitive to magnetic and microwave radiations. In both cases, the Fe_3_O_4_ core functioned for magnetic targeting, allowing them, with the help of an external magnet, to concentrate the NPs in the desire tissue, while the ZnO shell acted as a microwave absorber that facilitated the release of the drug due to an increase in temperature. The first system consisted of Fe_3_O_4_@ZnO@mGd_2_O_3_:Eu@P(NIPAm-*co*-MAA) NPs used as drug carrier of the cytotoxic etoposide (VP-16). The mesoporous Gd_2_O_3_:Eu shells acted as drug nanocarrier, being the poly[(*N*-isopropylacrylamide)-*co*-(methacrylic acid)] polymer (P(NIPAm-*co*-MAA)) the temperature sensitive caps that responded to microwave application. The experiments demonstrated that the ZnO shells effectively absorbed and converted microwave irradiation to heat; as a result, P(NIPAm-*co*-MAA) contracts, unblocking the mesopores and triggering the release of about 81.7% of the entrapped VP16 drug within 10 h [54]. The second nanoprobes were core–shell structured β-CD-Fe_3_O_4_@ZnO:Er^3+^,Yb^3+^ nanoparticles and their scheme of synthesis is shown in Figure 4a. In this system, the drug was stored in the inert cavity of the β-cyclodextrins (β-CD) due to hydrophobic interactions. The ZnO shell doped with Er^3+^ and Yb^3+^ not only acted as microwave absorber that produd a thermal response (similar to the previous one) but also provided fluorescence imaging for in vitro detection. The data showed that β-CD-Fe_3_O_4_@ZnO:Er^3+^,Yb^3+^ NPs were able to transform the microwaves into localized internal heating, allowing the release of the drug in a control manner by selecting the microwave exposure time and the number of cycles applied (Figure 4b). The MTT assay showed that the NPs had a strong targeting effect producing high rates of tumour cell death almost without affecting healthy ones [55].

### 2.4. ZnO QDs as Pore Caps

**Figure 5:**
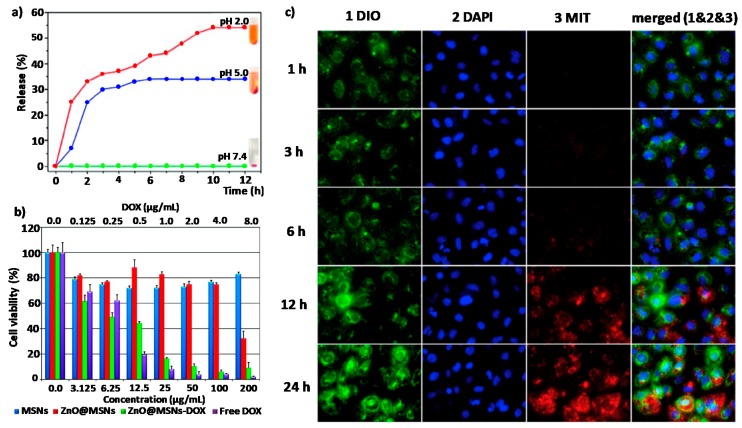
(**a**) DOX release profiles from ZnO@MSNsDOX at 3 different pH values (7.4, 5.0, and 2.0); (**b**) In vitro viability of HeLa cells in the presence of COOHMSNs, ZnO@MSNs, ZnO@MSNsDOX, and free DOX. Reproduced with permission from [56]. American Chemical Society, 2011; (**c**) Confocal microscopy images taken at different times of A549 cells incubated with the MIT-loaded, ZnO-gated MCNs. Cell membranes were stained in green, cell nuclei were stained in blue and released drugs wre presented red. Reproduced with permission from [60]. Elsevier, 2016.

ZnO QDs are sensitive to pH and can be synthesized at different sizes. These two features make them great candidates to act as “gatekeeper” of the pores of bigger systems, allowing to enclose in its interior different drugs that will only be released under acidic tumour conditions after dissolution of the QDs. Based on this idea, the mesoporous silica nanoparticles with a large load capacity and a pore diameter around 2.5 nm seem to be the perfect combination [66,67,68]. In fact, several groups have proposed different systems based on the combination of these two components, mesoporous silica nanoparticles as storage material and ZnO QDs as cap of the pores. It was in 2011 when G. Zhu et al. reported this combination for the first time. They synthesized MSN with amino groups inside the pores and carboxylic acid groups outside and loaded them with DOX. Finally, they used amino functionalized ZnO nanolids (Nls) to close the pores through amide coupling with 1-ethyl-3-(3-dimethylaminopropyl) carbodiimide (EDC). The stimulus-responsive capacity of the DOX-loaded ZnO MSNs were studied at different pH values. At physiological pH (7.4), negligible DOX release was observed; however, at pH 5.0m a fast DOX release was observed due to the dissolution of the ZnO Nls (Figure 5a). As can be seen in Figure 5b, the viability studies demonstrated that ZnO MSNs@DOX led to high death rates in HeLa cells at very low concentrations (6.25 μg/mL) [56].

Two years later, the same group presented a new system whose novelty lay in the fact that ZnO QDs were also used as chelate forming agents of a second hydrophobic drug (curcumin) that was significantly loaded onto the surface of the ZnO Nls. Cell viability experiments confirmed that the combination of both drugs presented a high cytotoxic effect even for small device concentrations such as ~3 μg/mL [57]. In 2015, H. Zhang et al. came out with a multifunctional nanotheranostic agent with lanthanide-doped upconverting nanoparticles (UCNPs) (NaYF_4_: 20%Yb^3+^, 2%Er^3+^/NaGdF_4_: 2%Yb^3+^) as the core for the UCL/CT/MRI trimodality imaging. In this system, called UCNPs@mSiO_2_-ZnO, the core was cover with a mesoporous silica layer, loaded with a drug and finally pH-sensitive ZnO QDs were used to cap the pores. UCNPs@mSiO_2_-ZnO demonstrated great abilities to be employed as contrast agents for tri-modal imaging, both in vitro and in vivo. The cytotoxic effect of the loaded NPs was studied against HeLa cell and the results showed higher therapeutic effectiveness than that obtained with the free DOX [58]. Lei Sun et al. prepared a new protease/redox/pH triple-sensitive delivery nanodevice through the combination of fluorescent ZnO QDs, oxidized glutathione (GSSG) and amino-functionalized pSiO_2_ NPs. In that case, GSSG was linked to the surface of NH_2_-functionalized pSiO_2_ NPs through amido bonds. Then, ZnO QDs were also covalently attached to GSSG acting as fluorescence probes and caps of the pores. In that system, the release of the amoxicillin cargo was susceptible to being triggered by three different stimuli: (i) the degradation of ZnO QDs in acid media characteristic of tumour environments; (ii) the break of the GSSG linkers through amido bonds by proteases; or (iii) the break of the GSSG linkers through disulphide bonds by the presence of reduced glutathione (GSH). Three different release experiments were performed to study the effect of each of the stimuli separately. It was observed that the amoxicillin release was slower because of GSH than that in the acidic pH conditions, which authors attribute to the presence ZnO QDs hindering GSH from entering the mesoporous structure to break the disulphide bonds. However, this dependency showed that the addition of protease K was higher, achieving around 66% of cargo release in only 6 h. The authors expected that this triple-stimuli responsive nanodevice had great applications for antitumour therapy [59]. One year after, in 2017 the same research group enhanced the GSH response of the nanodevice by anchoring the ZnO QDs through cystine (Cys) molecules and studied the in vitro cytotoxic effect of the system against HepG_2_ cells. After incubation for 48 h without drug cargo, the cell viability was about 96.6%, even at a high concentration of 500 µg/mL, which demonstrates that both l-pSiO_2_/Cys and l-pSiO_2_/Cys/ZnO presented good biocompatibility. However, when the same experiment was carried out, the DOX-loaded l-pSiO2/Cys nanoparticles displayed a concentration-dependent cytotoxicity significantly higher than that observed for free DOX [25]. Mesoporous carbon nanoparticles (MCNs) present similar characteristics to those described for MSNs in terms of mechanical and structural properties. That is why X. Du et al. reported a new carboxylated MCNs were ZnO QDs were covalently linked via dual amide bonds to minimize premature release of mitoxantrone (MIT) drug. The toxicity of the system itself was studied in vitro in A549 cells showing low toxicity after 48 h of incubation. However, when ZnO-gated MCNs were loaded with MIT the tumour killing capability was clearly increased to values of about 65% even at small drug concentrations such us 0.066 ng/cell. As can be seen in Figure 5c, the release of the drug was monitored by confocal microscopy, showing that MIT was exclusively released inside the cells without premature release in the extracellular media. This proved that the release is due to the dissolution of the ZnO QDs by a decrease in the pH inside the cell [60].

### 2.5. ZnO QDs That Provides an Added Value to Other Systems

**Figure 6:**
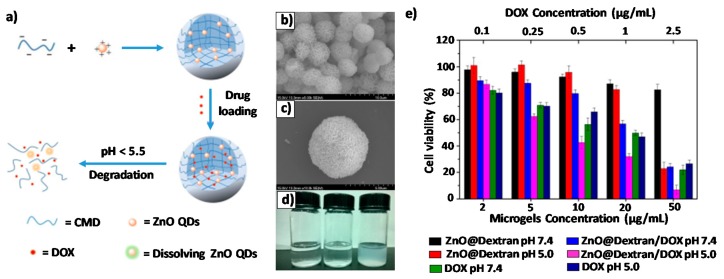
(**a**) Schematic representation of fabrication and degradation process of the ZnO@Dextran microgels; (**b**,**c**) Two different magnification SEM photographs of ZnO@Dextran microgels; (**d**) Digital photos of ZnO@Dextran microgels after incubation at pH 3.0 (left), 5.0 (middle) and 7.4 (right); (**e**) Cell viabilities of HeLa cells after being incubated with different samples under different conditions. Reproduced with permission from [61]. John Wiley and Sons, 2018.

Microgels are three-dimensional networks which have various applications due to their facile fabrication, their drug loading capacity and the possibility to functionalize them to obtain stimuli responsive effect. There are two strategies to achieve these stimuli responsive microgels: utilizing sensitive polymers and designing microgels with degradable crosslinkage. The latter strategy was used by J. Feng et al. to design ZnO@Dextran microgels loaded with DOX, were amino-modified ZnO QDs acted as pH-sensitive crosslinkers to carboxymethyl dextran (CMD)(Figure 6a–c). Since ZnO QDs are dissolvable at pH lower than 5.5, degradation of the ZnO@Dextran microgels at different pH values (7.4, 5.0, and 3.0) was investigated. As shown in Figure 6d, the digital photos were taken after vortexing of the microgels in different buffers for several minutes. It was observed that the hybrid microgels dissolved completely at pH 5.0 and 3.0, yielding a clear solution but in the case of pH 7.4 a non-transparent solution could be seen, indicating that most of the microgel particles remained intact. To evaluate tumour therapeutic effect of the DOX-loaded ZnO@Dextran microgels, the cytotoxicities of ZnO@Dextran/DOX and the two control materials (ZnO@Dextran microgels and free DOX) were investigated by the authors in Hela cells by MTT assays. By an unusual procedure, the cells were incubated with one of those materials under pH condition of 5.0 and 7.4 for 24 h, respectively. As shown in Figure 6e, ZnO@Dextran/DOX showed more pronounced cytotoxic effects on Hela cells at pH 5.0 than 7.4 [61].

## 3. ZnO Nanoplatforms for Bacterial Infection

The increase in the quality of life has been associated with an increase in the longevity of the population and, therefore, to the number of fragile and immunocompromised people, who are candidates to suffer a bacterial infection. In addition, despite aseptic surgical techniques, the number of surgical interventions that are practiced each year is increasing, making it the appearance of a bacterial infection associated with implants even more possible.Thus, bacterial infections have attracted great attention from both the medical community and public worldwide [69]. There are several obstacles that hinder the effectiveness of bacterial infection treatments: (i) the lack of early stage detection methods [70], which in clinic are currently limited to indirect imaging modalities; (ii) the overuse of antibiotics favouring the appearance of bacteria resistant to multiple drugs, leading to antibiotic ineffectiveness [71]; (iii) biofilm formation, which has an extracellular matrix that makes it resistant to antibiotics [72]; amd (iv) intracellular infections, whose difficulty lies in the wide variety of mechanisms used by bacteria to ensure their survival such as inhibition of the phago/lysosome fusion, resistance to lysosomal enzymes attack, etc. [73].

Thus, it would be necessary to develop clinical imaging tools able to selectively attack invasive bacteria and/or biofilm, allowing early detection [74] and that at the same time can release some antibiotics or produce some therapeutic effect in the infected area [73]. Luminescent ZnO QDs, with high surface-to-volume ratio, capacity of ROS production and a strong surface chemistry, meet all the necessary requirements to adequately treat bacterial infection [75]. Besides, recent studies evidence that NPs can present antibacterial capacity without having any side effect on human cells, that is, showing selective toxicity [76]. This selectivity might be because of differences in the action mechanisms in bacteria and mammal cells. For bacteria, the most recognized NPs action mechanisms so far are ROS and free radical generation, bacterial cells adhesion and penetration, biofilm penetration and changes on bacteria metabolic activity and gene expression [77].

It has become clear that biofilm-grown cells exhibit different properties than planktonic bacteria, therefore nanodevices with different properties need to be use for the treatment of each one [78].

### 3.1. ZnO Nanoplatforms for Planktonic Bacteria Treatment

ZnO QDs have proven their effectiveness against a broad spectrum of pathogenic microorganisms, even though the response they produce in Gram-positive/-negative studies are different, probably due to differences in the membrane composition and in the intracellular antioxidant content of both types of bacteria [7]. It has also been reported that the antimicrobial capacity of the ZnO clearly depends on the size of the QDs, i.e., with smaller sizes being more effective [79].

The antibacterial effect of ZnO QDs on *Campylobacter jejuni* was investigated by X. Shi et al. for inhibition and inactivation of cell growth. In this study, the effect of ZnO QDs on the integrity of the bacterial membrane and on the expression of different bacteria genes was studied. Ethidium monoazide (EMA) is a fluorescent label able to bind DNA and inhibit PCR amplification. However, the bacterial membrane is impermeable to EMA so the union only take place if the membrane has been damaged. EMA-PCR experiments were carried out and a clear reduction on DNA amplification was observed for the samples treated with ZnO QDs. Besides an increase expression of ahpC and katA, two common genes that express oxidative stress, was also observed. These PCR results suggested that the antibacterial mechanism of ZnO nanoparticles in Campylobacter was most likely due to disruption of the cell membrane and oxidative stress [80]. I. Ahmad et al. performed a comparative experiment to study the antibacterial activity of ZnO QDs against different types of bacteria, including some Gram-negative (*Vibrio cholera*, *Campylobacter jejuni* and *Escherichia*) and Gram-positive (methicillin resistant *Staphylococcus aureus*) studies. After 4 h of ZnO QDs exposure, a clear decrease in bacterial viability was observed in all cases, being especially effective against *C. jejuni* where bacterial death amounted to 65%. On the other hand, *V. cholerae* were the least affected bacteria seeing its population reduced by only 27% [75]. S. Arastoo et al. synthesized Ag and ZnO QDs and mixed them to study their antibacterial effect against *Mycobacterium Tuberculosis* (Mtb) growth inside macrophages. The toxicity of the QDs in THP-1 leukemic cells was also studied. Results showed that the 8ZnO/2Ag ratio was the most effective since it had high antibacterial capacity against Mtb both in vivo and in vitro but without affecting the cellular viability of THP-1 cells [81]. In 2016, R. Jalal et al. used different techniques (fluorescence and scanning electron microscopy, flow cytometry and DNA extraction) to elucidate the mechanism of action of ZnO QDs administered alone and in combination with two antibiotics ciprofloxacin and ceftazidime. The authors demonstrated that sub-inhibitory concentrations of ZnO QDs were enough to significantly increase the uptake and antibacterial capacity of both antibiotics.

The effect of light on increasing ROS production for the treatment of bacteria had also been studied. J. Gupta and D. Bahadur reported the antibacterial and anticancer activity effect of visible light irradiation on Cu substituted ZnO nanoassemblies (Cu-ZnO NAs). First, different concentrations of Cu were studied to optimize the ROS production with Cu_5_-ZnO being the most efficient material. The data showed that the NAs themselves had antibacterial activity. Although irradiation with visible light alone had a negligible effect on cell viability, the combination of light with the Cu5-ZnO NAs produced 100% of bacterial death after 30 min of light application [82].

**Figure 7:**
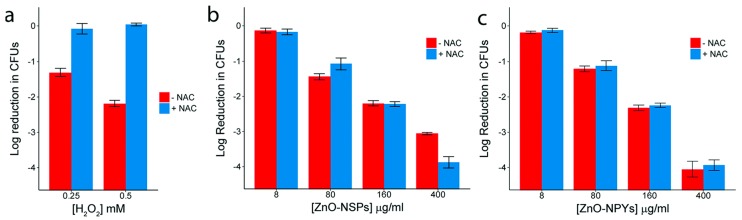
Reduction in colony counts (from 5 × 10^7^) observed after exposure to increasing concentrations of: (**a**) H_2_O_2_; (**b**) ZnO-NSPs; or (**c**) ZnO-NPYs, with and without 50 mM NAC. Reproduced with permission from [83]. Royal Society of Chemistry, 2018.

Opposite to what has been said so far, VanEpps et al. have recently published an article that casts serious doubts on ROS production being responsible for the antibacterial efficacy of ZnO QDs. They based their conclusions on experiments made with two different types of ZnO nanoparticles against *S. aureus* bacteria. Bacteria were exposed to both types of NPs and concentrations of H_2_O_2_ that presented an equivalent amount of bacterial killing capacity. Additionality, the same experiment was carried out but in presence of the antioxidant N-acetylcysteine (NAC). Results showed a clear recovery of those bacterial colonies treated with H_2_O_2_ but no changes in the ones treated with ZnO laying bare that the decrease in bacteria population was not due to ROS production (Figure 7).

Studies on the effect of ZnO NPs on gene expression were consistent with that as oxidative stress genes were down regulated. In addition, the effect on anaerobic carbohydrate metabolism and energetics with upregulation of the UMP biosynthesis pathway was also proposed as the reason for ZnO NPs antibacterial activity [83].

As in the case of cancer, the use of ZnO nanosystems for the treatment of bacterial infections is not only limited to QDs themselves, but ZnO QDs can also be used as a component of more complex systems. In 2015, Parkin et al. incorporated crystal violet and zinc oxide nanoparticles (CVZnO) into medical grade polyurethane polymers to synthesize new surfaces with antibacterial activity. The antibacterial capacity of these surfaces after light irradiation proved to be lethal for the two types of bacteria studied: *E. coli* and *S. aureus*, for Gram-negative and Gram-positive, respectively [84]. One year later, Gu et al. developed a fluorescent nano-probe MPA@ZnO-PEP by the combination of silica stabilized ZnO QDs (ZnO@SiO_2_) with PEP, a peptide fragment for bacteria targeting, and MPA, a near infrared dye. This fluorescent nanodevice proved to be a potent tool to bacteria detection both in vitro and in vivo since it allowed to differentiate between sterile inflammation and bacterial infection. To increase the antibacterial capacity, vancomycin (Van) was also incorporated to the nanoplatform to form the so-called MPA/Van@ZnO-PEP. Antibacterial (*S. aureus* and *B. subtilis*) test were performed, showing that for *B. subtilis* a concentration of 1mg was enough to produce bacterial inhibition, needing 2 mg to achieve the same effect in *S. aureus*. In both cases, it was a much lower concentration than that required for free Van. Furthermore, authors wanted to test the efficacy of the nanosystem against antibiotic resistant bacteria. Therefore, Van was substituted by methicillin (Met) and the antibacterial capability of MPA/Met@ZnO-PEP was studied against to a *S. aureus* strain which was resistant to this antibiotic (MRSA). While bacteria incubated with free Met or the unloaded nanoplatform exhibited a viability comparable to the control, those incubated with MPA/Met@ZnO-PEP reduced their viability to a 60% for a concentration of 64 µg/mL of NPs [85].

### 3.2. ZnO Nanoplatforms for Biofilm Treatment

Biofilms are defined as complex communities of microorganisms that grow embedded in a self-produced protective extracellular matrix made of polysaccharides, DNA and proteins. The main problem related with biofilm formation is that at this point bacteria becomes antibiotic resistant and more tolerant to disinfectant chemicals or to the response of the immune system, increasing the probability to degenerate into chronic infections [72]. Clinical observations and experimental studies clearly indicate that a treatment exclusively based in the administration of antibiotics is usually insufficient to eliminate the biofilm. Hence, to effectively eradicate biofilm infections, new, more complex strategies such as the use of ZnO nanodevices are needed.

**Figure 8:**
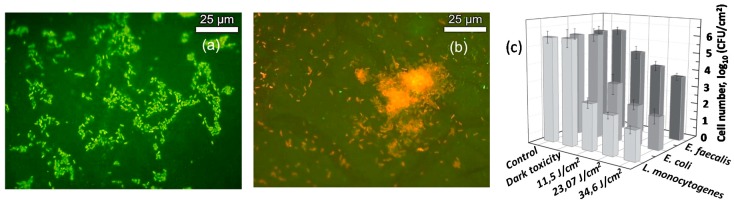
Fluorescence micrographs of *E. coli* biofilms grown up onto ZnO NRs surfaces: (**a**) without; or (**b**) with light treatment. Alive cells were stained in green and dead cells in red; (**c**) Light-dose dependence cytotoxic effect on *E. faecalis* MSCL 302, *L. monocytogenes* ATCL3C 7644 and *E. coli* O157:H7 biofilms grown up onto ZnO NRs coated surfaces. Non-illuminated biofilms grown on plastic surfaces were used as control and effect the NRs in absence of light was expressed as dark toxicity. Reproduced with permission from [86].

Juršėnas et al. focused their research on the effect of unmodified ZnO nanorods (NRs) against strongly resistant bio-films after 405 nm light excitation. Therefore, different bacteria biofilms were grown onto surfaces covered by ZnO NRs. Then some of them were exposed to light while the others remained in darkness. The results showed that neither the NRs nor the irradiation with light presented by themselves any type of toxicity against planktonic bacteria or biofilms (see Figure 8).

However, for those samples coated with ZnO NRs and subject to light, the biofilm was substantially reduced. Besides, it was observed that inactivation of biofilm growth was strongly dependent on light dose. Moreover, different degrees of effectiveness were observed for the same treatment depending on the type of bacteria used, with *L. monocytogenes* biofilms being the most susceptible population and *E. faecalis* the most resistance one [86].

### 3.3. ZnO Nanoplatforms for Planktonic and Biofilm Treatment

The ultimate treatment would be one that involve a simultaneous attack of the planktonic bacteria and the undesired bacterial biofilm. Recently, several systems based on ZnO QDs have been shown to fulfil this double functionality. An example of this was the research carried out by A.A. Al-Khedhairy et al. about the effect of ZnO QDs against bacterial strain *Pseudomonas*. For the study of the effect on bacteria, several plates were cultured with increasing QD concentrations, observing a maximum inhibition at 100 mg/mL of ZnO QDs. To follow the inhibition of biofilm formation, crystal violet dye was employed as stain. The results showed a significant inhibition of the biofilm formation at 50 and 100 mg/mL of ZnO QDs [87]. J. Lee et al. also used *Pseudomonas aeruginosa* as bacteria but in this case the effect of thirty-six metal ions was studied. Among all of them, zinc ions and ZnO QDs were able to efficiently reduce *P. aeruginosa* biofilm as well as the production of different bacterial growth signals [88]. Similarly, A. Jamalli et al. studied the effect of ZnO QDs in the biofilm formation and the antigen 43 expression (an important surface protein in *E. coli* which is encoded by flu gene). The authors observed that ZnO QDs produced inhibitory effects on biofilm formation in uropathogenic *Escherichia coli* (UPEC) isolates. Results also showed that concentrations of QDs lower than the amount needed to inhibit biofilm formation were nevertheless enough to significantly decrease the expression of the flu gene in UPEC [89].

**Figure 9:**
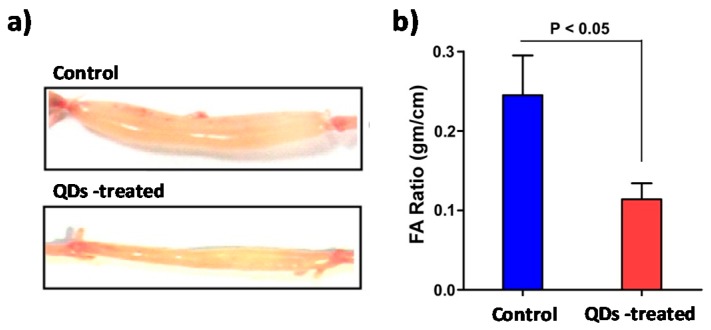
(**a**) Image of isolated mouse intestinal loop 6 h after the injection of 1 µg of CT and 1.25 µg µL^−1^ of ZnO QDs or only CT in the control one; (**b**) Fluid accumulation (FA) ratio after 6 h of injection; (n = 10 mice with 15–20 loops studied per group). Reproduced with permission from [90]. Elsevier, 2016.

Chakrabarti et al. studied the effect of ZnO QDs on two biotypes of cholera bacteria (classical and El Tor) observing greater efficiency for El Tor strain both in biofilm and in planktonic forms. The authors were not completely sure about the reason for the differences in susceptibility. However, according to their suggestion, it might be due to some differences in membrane structure or gene expression between the two biotypes. Results showed that ZnO QDs produced ROS that damaged bacterial membrane and substantially modified their morphology. Authors also tested the antibacterial capacity of the QDs in cholera toxin (CT) mouse models. As can be seen in Figure 9a, controls presented strong fluid accumulation in the loops and therefore a big organ distention and diarrheal symptoms.

Fluid accumulation values equal to 0.2 or higher are considered a positive result in diarrhoea. Figure 9b shows that a single administration of ZnO QDs with CT was enough to considerably reduce intestinal fluid accumulation. The synergist effect of the combine administration of ZnO QDs and kanamycin was also studied achieving a considerably increased killing capability of 85–87% killing compared with 50–70% of the QDs alone [90].

Another important contribution of ZnO against infection is its use as coating on implant materials. In 2016, Rose et al. reported the effect of three different ZnO QDs structures (spheres, plates and pyramids) in planktonic growth and biofilm formation after surface coating. Several bacterial types were used, and planktonic growth experiments revealed that *S. epidermidis* and *S. aureus* (Gram-positive bacteria) presented dose dependent reduction for all ZnO structures. However, *K. pneumonia* and *E. coli* (Gram-negative bacteria) were not affected by the presence of ZnO QDs up to 667 μg/mL, probably because of differences in bacterial surface hydrophobicity.

Biofilm formation experiments on polystyrene ZnO QDs coated surfaces demonstrated no significant differences related with the particle morphology. Nevertheless, notable differences were observed in terms of the type of bacteria studied allowing a reduction in biofilm formation only for Gram-positive bacteria [91]. De Fátima Montemor et al. studied the antibacterial and antibiofilm effect of nano-and micro-sized ZnO coatings against MRSA. Which found that only the nano-sized coating was able to reduce biofilm formation. Additionally, the combination effect of ZnO coating followed by the addition of different antibiotics in sub-inhibitory concentrations was also analysed. The results showed a greater antibacterial efficacy due to the combine treatment with gentamicin, no differences when adding trimethoprim and even an infection worsening in the presence of rifampicin, ciprofloxacin, and vancomycin [92]. Recently, Wang et al. reported a new method to coat implant surfaces based on the combination of two different ZnO structures named as ZnO nanorods−nanoslices hierarchical structure (NHS). Authors modified the surface of two commonly used implant materials (titanium and tantalum) with NHS and each of the structures separately and studied their antibacterial capacity against *E. coli* and *S. aureus* under different times. Results proved that ZnO nanoslices rapidly released (48 h) allowing to kill bacteria in early stages. On the other hand, ZnO nanorods that presented higher stability needed around two weeks to present a bacteria killing capability. ZnO NHS, which combined both elements presented a two-stage antibacterial effect. The antibacterial efficacy of ZnO NHS was also studied by mice test in vivo, showing not only good results onto the implant surfaces but in the surrounding areas where an effective sterilization was also observed [93]. 

However, despite the numerous advantages of the antibacterial character of ZnO QDs, a poor control of their residues can have negative effects on soil-dwelling microorganisms responsible for numerous activities of great impact on the soil quality such as plant protection, biodegradation, ecological balance and nutrient recycling [94].

All recent advances made for the treatment of bacterial infection with ZnO nanoplatforms are summarized in Table 2.

## 4. Antifungal Capacity of ZnO Nanoplatforms

The antimicrobial power of the ZnO QDs is not limited to bacteria but also to other types of microorganisms such as fungi. The advantage of these materials is that their effectiveness can be applied to treat different problems caused by microorganisms such as, infections, diseases, biocontamination and corrosion.

**Figure 10:**
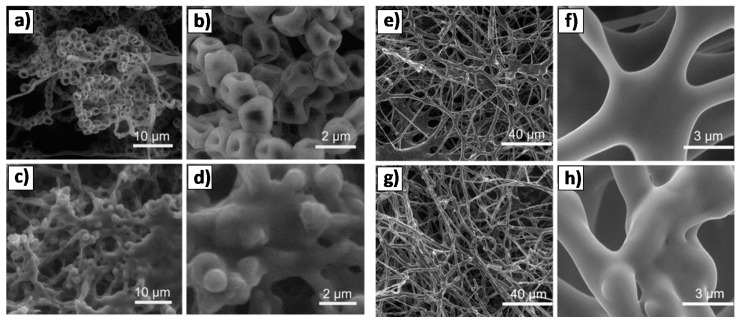
SEM images of (**a**–**d**) *Penicillium expansum* and (**e**–**h**) *Botrytis cinerea* without (**a**,**b**,**e**,**f**) or with (**c**,**d**,**g**,**h**) the treatment of ZnO QDs suspension. Reproduced with permission from [97]. Elsevier, 2011.

Growth of fungal pathogens is one of the main problems in agriculture and usually causes high economic losses to farmers [96]. Different groups have reported the antifungal efficacy of ZnO QDs against plant pathogens. M. Lin et al. observed that ZnO QDs at concentration higher than 3 mmol/L could markedly reduce the growth of *B. cinerea* getting even better results against *P. expansum* (Figure 10) [97].

The antifungal effect of ZnO QDs has also been proven against other plant pathogens [98,99,100].

Another important problem related with microorganism and especially with fungi is the biological colonization of stone heritage what gives rise to its biodegradation. Recently, P. Quintana et al. studied the antifungal and photocatalytic properties of ZnO, MgO, and Zn/Mg Oxide QDs for the protection of calcareous stone heritage. Results showed that the growth inhibition of *Penicillium oxalicum*, *Pestalotiopsis maculans*, *Paraconiothyrium* sp., and *Aspergillus niger* fungi achieved by Mg_1−x_Zn_x_O QDs was higher than that obtained by either of the two pure oxide QDs. The photocatalytic activity observed was also higher in the case of the mixed oxides compare with the pure ones [101].

The infection in humans due to the action of opportunistic fungi is also a relevant health problem nowadays. N.O. Jasim reported the effect of ZnO QDs against two different opportunistic fungi (*A. fumigatus*, *C. albicans*) and observed a significant decreased (*p* ≥ 0.05) in the radial growth of the fungus at different concentrations and an increase in the inhibitory effect when increasing the period of incubation [102]. To increase the antifungal effectiveness against *Candida albicans*, Ding et al. designed ZnO QDs coated by chitosan (CS) and functionalized with linoleic acid (LiA). Chitosan has been reported to present antimicrobial activity because of the presence of positive charges that react with cellular DNA of some bacteria [103]. Additionally, LiA with Trans-11 and Cis-9 arrangement is one of the effective acids on fungal growth [104]. The authors studied the effect of CS-LiA QDs compared with that produced by fluconazole (a potent antifungal drug). Results showed that CS-LiA QDs could inhibit the growth of fluconazole-resistant clinical strains at similar concentrations to fluconazole, but the inhibition percent of biofilm formation, for nanoparticles was greater than that of fluconazole [105]. The use of ZnO QDs to synthesize antifungal filters [106] or as antifungal bioglasses [107] for medical implants or surgical equipment has also been reported as a probe into the versatility of these QDs in the fight against microorganisms. All ZnO based nanoplatforms for antifungal treatment cited in this manuscript are summarized in the following Table 3.

## 5. ZnO Nanoplatforms for Diabetes Treatment

According with the National Diabetes Statistics Report of 2017 [108], 30.3 million people of all ages, or 9.4% of the U.S. population, had diabetes in 2015 and, unfortunately, the number of people suffering from this disease continues to grow over time. These figures make diabetes one of the greatest diseases facing our society today. In 1934, when it was observed that insulin crystal contained zinc [109], researchers began to believe that Zn, insulin and diabetes could be intimately related. Nowadays, it is known that Zn interacts with some elements of insulin signalling pathway and therefore affects the glucose metabolism. Besides, other aspects of diabetes disease such as β-cell function, glucose homeostasis, insulin action, and diabetes pathogenesis are also influenced by the presence of this ion [110]. As the degradation of ZnO results in the release of Zn^2+^ ions in the media, the number of studies about the incidence of ZnO QDs in the treatment of diabetes has increased in recent years. Umrani and Paknikar tested the antidiabetic effect of ZnO QDs in rats and observed that after oral administration the glucose tolerance was improved. Other factors were also enhanced such us a reduction in non-esterified fatty acids, triglycerides and glucose in blood and an increase in serum insulin [111]. In 2015 Asri-Rezaie et al. performed a comparative studied of the antidiabetic activity and toxic effects of ZnO QDs and zinc sulphate (ZnSO_4_) in diabetic rats. It was observed that those treated with ZnO showed greater antidiabetic activity compared to ZnSO_4_. However, severely elicited oxidative stress particularly at higher doses was also observed [112]. The efficacy of ZnO QDs in attenuating pancreatic damage in a rat model of treptozotocin-induced diabetes was also settled one year later by Kandeel et al. [113]. Other comparative studies including ZnO QDs for antidiabetic studies have been performed [114]. In 2016, Abu-Risha et al. went a step further and studied the antidiabetic effect of a co-administered treatment based on ZnO QDs and Vildagliptin, a standard antidiabetic drug. As a result, the recovery of the function and structure of β cells and a synergistic effect on the therapy of Type-2 diabetes between this two components was observed [115]. S.N. Kale et al. also explored the combination effect between ZnO and drugs but in this case the antidiabetic agent was conjugated to the surface of the QDs. Red Sandalwood (RSW), a potent natural extract anti-diabetic agent was chosen as model drug. Results showed that ZnO-RSW QDs was more effective against the crude murine pancreatic glucosidase than any of the two elements (RSW and ZnO QDs) that composed it administered separately [116]. All ZnO based nanoplatforms for diabetes treatment cited in this are summarized in Table 4.

## 6. ZnO Nanoplatforms with Anti-Inflammatory Properties

The beneficial health effects of both elemental zinc and its salts have been known for a long time, which is why for several hundred years they have been widely used for therapeutic purposes, including the anti-inflammatory effectiveness in several common inflammatory dermatoses and for wounds or ulcers [117]. Since the appearance of nanoparticles, and considering these beneficial properties of zinc ions, the anti-inflammatory capacity of ZnO QDs have also been studied. H. Alenius et al. investigated the response produced by topically administration of nano-sized ZnO (nZnO) in the mouse model of atopic dermatitis (AD) and compared these outcomes to those induced by bulk-sized ZnO (bZnO). Their experiments clearly demonstrated that nZnO efficiently reduced the thickness of the skin in the allergic environment compared with the effect produced by bZnO. While topical application of bZnO favoured macrophage infiltration, the recruiting of CD8^+^ and CD4^+^ T cells in allergic skin was strongly inhibited by nZnO treatment. These results demonstrated that topical nZnO treatment had great effects in reducing skin inflammation as consequence of allergen exposition [118].

**Figure 11:**
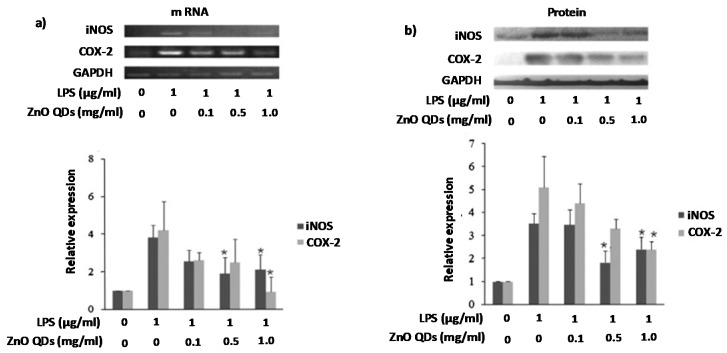
(**a**) Determination of iNOS and COX-2 mRNA levels by RT-PCR; (**b**) Determination of iNOS and COX-2 protein levels by Western blot (mean ± SD of n = 3 and * *p* < 0.05 versus LPS alone). Reproduced with permission from [119]. Elsevier, 2015.

The anti-inflammatory capacity of ZnO QDs is not limited to skin problems but has also proven to be very effective for other inflammatory diseases. Shin et al. studied the anti-inflammatory properties of ZnO in RAW 264.7 murine macrophage cells by measuring their effect in pro-inflammatory mediators. As can be seen in Figure 11, ZnO QDs clearly reduced inflammation and showed a dose-dependent effect in the suppression of different protein expressions (COX-2, iNOS, TNF-α and interleukins-1β and -6) and mRNA [119].

Inflammatory bowel diseases are widespread inflammatory diseases that cause debilitating health problems including cancer. In their research, Feng et al. showed that ZnO QDs treatment had markedly dose-dependent effects on the remission of dextran sulphate sodium influenced ulcerative colitis in mice. They also demonstrated that the antioxidant and anti-inflammatory abilities of ZnO QDs were related to their capacity to suppress ROS and malondialdehyde production; increase GSH level; suppress pro-inflammatory cytokines IL-1β and TNF-α and myeloperoxidase (MPO) [120]. Throughout this manuscript the ability of ZnO QDs to produce ROS has been highlighted. However, the anti-inflammatory properties of these particles are now related to their ability to eliminate them. Although this may seem contradictory, the truth is that the same properties (large surface area and high catalytic capacity) that favour the generation of ROS in environments where they are not present is probably responsible for suppressing them in environments where they are very abundant. Ulcerative colitis tissues had 10- to 100-fold increased ROS, and in this case, the antioxidant activity of ZnO QDs might be the result of electron density transfer from the oxygen to the odd electron placed on outer orbits of oxygen in O_2_^•^^−^ and OH^•^ radicals [121]. Selected ZnO based nanoplatforms with anti-inflammatory properties are presented in Table 5.

## 7. ZnO Nanoplatforms for Wound Healing

**Figure 12:**
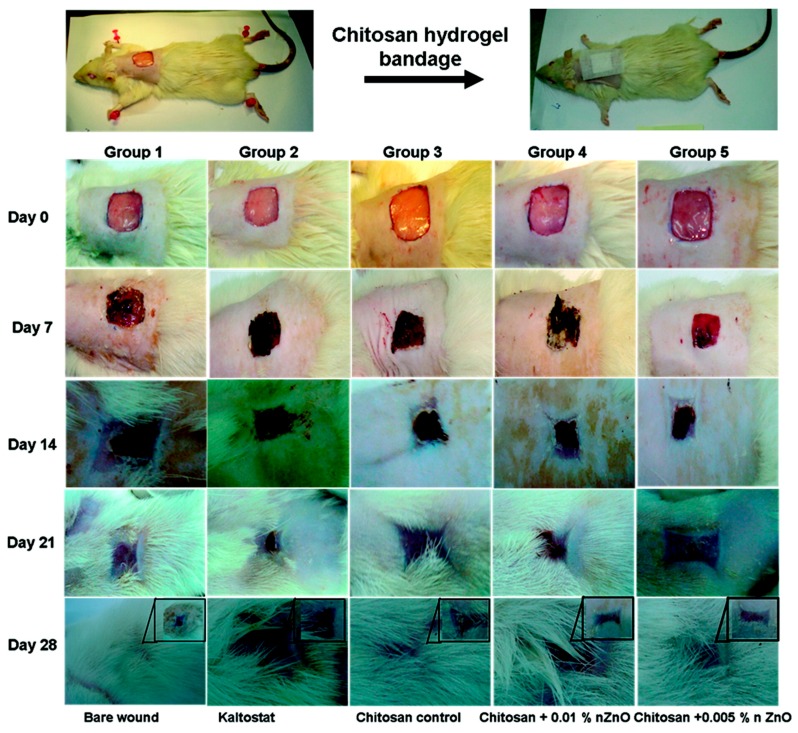
In vivo images of the wound healing process in Sprague−Dawley rats. Reproduced with permission from [125]. American Chemical Society, 2012.

The lack of zinc production delaying wound healing is a fact well known in clinic. Already in 1990, the effect of topically applied zinc on leg ulcer healing and its effect on some mechanisms in wound healing was studied using standardized animal models [122]. After that, several clinical and experimental studies were performed with elemental Zn and zinc oxide [123]. Results showed that treatments based in topical ZnO application produced several benefits in re-epithelialization, wound healing, infections and ulceration among others [124]. Currently, researchers are mostly focusing their efforts on the design of new materials for wound healing that incorporate the antibacterial and healing effects of ZnO QDs. R. Jayakumar et al. synthesized a microporous chitosan hydrogel based on ZnO composite bandages (CZBs) that presented great flexibility. In addition, ZnO QDs were incorporated into a hydrogel made of chitosan. Results showed that these nanocomposite bandages presented antibacterial activity and great effects on enhancing blood clotting and swelling capacity. Infiltration and cell attachment studies were performed showing a clear penetration and nanocomposite attachment of the cells. Furthermore, as can be seen in Figure 12, the in vivo test in Sprague-Dawley rats proved the efficacy of these bandages in wound healing as well as their beneficial effects on collagen deposition and better re-epithelialization [125].

Manuja et al. synthesized ZnO QDs-loaded-sodium alginate-gum acacia hydrogels (SAGA-ZnO QDs) by bounding the aldehyde and hydroxyl groups of gluteradehyde and sodium alginate polymer, respectively. Results showed that hydrogels were biocompatible in peripheral blood fibroblast/mononuclear cells and could produce inhibition of *Pseudomonas aerigunosa* and *Bacillus cereus*. SAGA-ZnO QDs hydrogels also presented a great healing capacity in sheep fibroblast cells even at low concentrations [126].

Due to its excellent properties including biocompatibility, haemostatic, bacteriostatic and healing capacity, chitosan has been extensively used in biomedicine [127]. In 2017, two research groups reported two different composites based on chitosan and containing ZnO QDs to combine antibacterial and wound healing properties in the same material. Denkbas et al. proposed the use of scaffolds containing chitosan/silk sericin (CHT/SS) in combination with lauric acid (LA) or ZnO QDs for wound dressing applications. Although both combinations CHT/SS/ZnO QDs and CHT/SS/LA had antimicrobial effect against *E. coli* and *S. aureus*, the presence of LA proved to be clearly more efficient for both bacteria. Besides, these nanocomposites were able to improve the attachment, proliferation and growth of HaCaT cells, presenting no secondary effects [128]. The second composite was proposed by Bezerra et al. and consisted in chitosan-based films that contained chondroitin 4-Sulphate, gelatin and zinc oxide nanoparticles. The wound healing capacity of the system was evaluated in rat models with full-thickness excision. After six days, results showed a significant decrease (14–35% more) of the wound compared with that obtained in control ones [129]. Selected ZnO based nanoplatforms for wound healing are summarized in Table 6.

## 8. Conclusions and Future Outlook

We demonstrate in this article of bibliographic review that zinc oxide nanoparticles present unique properties: (i) luminescence; (ii) Zn^2+^ ions release in aqueous media, especially in acidic conditions; (iii) ROS generation, mainly after being irradiated with UV light; (iv) versatile surface chemistry; and (v) an easy and economical synthetic process that allows an accurate control of the size and shape of the particles. All these factors make this nanomaterial a very versatile system, by itself or combined with other elements. This proves that ZnO nanodevices have applications in virtually all fields of science, especially in biomedicine. In this review, we report the latest advances derived from their used in the treatment of cancer and diabetes; their antibacterial, antifungal and anti-inflammatory efficacy; and their wound healing capacity. Despite all the advances in the understanding of ZnO nanostructures mechanism of action and biological effects, there is still a lack of knowledge on this subject, especially on what the long-term effects are. Therefore, a greater knowledge is necessary to assess whether the already multiple known advantages of the ZnO outweigh the potential risks. In addition, nowadays, reproducibility in the NPs synthesis process is still a challenge to be achieved. This, together with the lack of a standardized protocol (concentrations used, times of action, etc.) that allows comparing the different studies, makes it even more complex to obtain a complete knowledge of the NPs effects and therefore their clinical translation. However, in view of the facts, the opinion of the authors is that this is only the beginning and that, in the near future, ZnO based nanodevices will not only will achieve clinical states but other novel theranostic applications will also be proposed for their use.

## Figures and Tables

**Table 1:** ZnO nanoplatforms for theranostic in Cancer.

Type of Cell/Animal Used ^a^	Type of Device ^b^	Responsive Phenomena ^c^	Drug/Antibiotic ^d^	Reference
MCF-7	ZnO QDs	-	Adsorbed DOX	[42]
MCF-7R, MCF-7S	ZnO QDs	pH	Loaded DOX	[43]
MDA-MB-231, HeLa, NCI/ADR-RES, MES-SA/Dx5	ZnO QDs	pH	Adsorbed DOX	[44]
-	ZnO QDs	pH, ultrasounds	Loaded DOX	[45]
HeLa	FA Mg ZnO QDs	pH	Adsorbed DOX	[46]
MCF-7, MDA-MB-231, nude mice	FA Hollow ZnO NPs	pH	Loaded paclitaxel	[47]
MDA-MB-231, HBL-100, mice	FA ZnO Nanosheets	pH, heat	Loaded DOX	[48]
SMMC-7721	ZnO nanorod	UV radiation	-	[12]
HeLa, PC3	Lanthanide–ZnO QDs	UV, X-ray, γ-ray radiation	-	[33]
SMMC-7721	ZnO nanorod	UV radiation	DOX complex	[49]
HNSCC	ZnO QDs	UVA irradiation	Paclitaxel, cisplatin	[50]
MCF-7	MUC1 aptamer S2.2. ZnO QDs	UV radiation	Loaded DOX	[51]
BxPC-3, tumour-bearing nude mice	Gd-Polymer–ZnO QDs	pH	Adsorbed DOX	[34]
HEK 293T, HeLa	FA-SiO_2_ ZnO NPs	pH	Loaded DOX	[52]
HeLa	Lipid ZnO NCs	pH	-	[53]
Caco-2	TiO_2_@ZnO–GO and TiO_2_@ZnO	pH	Loaded Cur	[26]
-	Fe_3_O_4_@ZnO@mGd_2_O_3_:Eu@P(NIPAm-*co*-MAA)	Microwave, Magnetic radiation	VP-16	[54]
MCF-7	β-CD-Fe_3_O_4_@ZnO: Er^3+^, Yb^3+^	Microwave, Magnetic radiation	VP-16	[55]
HeLa	ZnO MSNs	pH	Loaded DOX	[56]
BxPC-3	Mg ZnO MSNs	pH	Loaded CPT, adsorbed Cur	[57]
HeLa, mouse	UCNPs@mSiO_2_-ZnO	pH	Loaded DOX	[58]
-	ZnO-pSiO_2_-GSSG NPs	Protease, redox, pH	Loaded amoxicillin	[59]
HepG_2_	l-pSiO_2_/Cys/ZnO NPs	Redox, pH	Loaded DOX	[25]
A549	ZnO-MCNs	pH	Loaded MIT	[60]
HeLa	ZnO@-Dextran microgels	pH	Loaded DOX	[61]

^a^ MCF-7: Human breast cancer cell; MCF-7S/MCF-7R: Human breast cancer cell sensitive/resistant to doxorubicin; MDA-MB-231: epithelial, human breast cancer cell; HeLa: Human epithelial cells from a fatal cervical carcinoma; NCI/ADR-RES: Ovarian tumour cell; MES-SA/Dx5: Multidrug-resistant human sarcoma cell; HBL-100: Human, Caucasian, breast cancer cell; SMMC-7721: Human hepatocarcinoma cell; PC3: Human prostate cancer cell; HNSCC: Head and neck squamous cell carcinoma; BxPC-3: Human pancreatic cancer cell; HEK 293T: Human embryonic kidney cells; Caco-2: Human epithelial colorectal adenocarcinoma cell; HepG_2_: Human liver cancer cell; A549: Adenocarcinomic human alveolar basal epithelial cell. ^b^ ZnO QDs: Zinc oxide quantum dots; FA: Folic acid; QDs: Quantum dots; MUC1: membrane glycoprotein which is highly expressed in most breast cancers; Aptamer S2.2.: (5′-COOH-GCA-GTT-GAT-CCT-TTG-GAT-ACC-CTGGTTTTT-FAM-3′) SiO_2_: Silica; NCs: Nanocrystals; MABG: TiO_2_@ZnO–GO: ZnO coated mesoporous titanium oxide QDs containing graphene oxide; Fe_3_O_4_@ZnO@mGd_2_O_3_:Eu@P(NIPAm-*co*-MAA): iron oxide QDs coated with ZnO and mesoporous Gd_2_O_3_:Eu shells with a polymer poly[(*N*-isopropylacrylamide)-*co*-(methacrylic acid)] (P(NIPAm-*co*-MAA)) to gate the mesoporous; K8(RGD)2 cationic peptide containing 2 RGD sequences; β-CD-Fe_3_O_4_@ZnO: Er^3+^, Yb^3+^: β-cyclodextrins functionalized iron oxide QDs doped with Er^3+^ and Yb^3+^ coated with ZnO; ZnO MSNs: Mesoporous silica nanoparticles with ZnO QDs as cap of the pores; UCNPs@mSiO_2_-ZnO: Lanthanide-doped upconverting nanoparticles with a mesoporous silica layer and ZnO QDs as gatekeeper; ZnO-pSiO_2_-GSSG NPs: ZnO QDs as cups of oxidized glutathione (GSSG) amino-functionalized silica NPs; l-pSiO_2_/Cys/ZnO NPs: Lemon like silica NPs with cysteine and ZnO QDs cups; MCNs: Mesoporous carbon nanoparticles. ^c^ UV: Ultra violet. ^d^ DOX: Doxorrubicin; Cur: Curcumin; VP-16: Chemotherapeutic drug etoposide; CPT: Camptothecin; and MIT: Mitoxantrone.

**Table 2:** ZnO nanoplatforms for theranostic bacterial infection.

Type of Bacteria Used ^a^	Type of Device ^b^	Responsive Phenomena ^c^	Drug/Antibiotic ^d^	Reference
*C. jejuni*	ZnO QDs	-	-	[80]
EPEC, *C. jejuni*, *V. Cholerae*, MRSA	ZnO QDs	-	-	[75]
THP-1, *M. tuberculosis*	ZnO QDs + Ag QDs	-	-	[81]
*A. baumannii*	ZnO QDs	-	Coadministered Cip, Cef	[95]
*E. coli*	Cu-ZnO NAs	Visible light	-	[82]
MRSA	ZnO-NPYs, ZnO QDs	-	-	[83]
*S. aureus*, *E. coli*	CVZnO polyurethane surface	White light	Loaded CV	[84]
*S. aureus*, *B. subtilis*, MRSA, *S. aureusor B*. *subtilis* infected mouse	MPA@ZnO-PEP	-	Loaded Met, Van	[85]
MSCL 302, ATC_L3_C 7644, O157:H7	ZnO NRs	UV light	-	[86]
*P. aeruginosa*	ZnO QDs	-	-	[87]
*P. aeruginosa*	ZnO QDs	-	-	[88]
*E. coli*	ZnO QDs	-	-	[89]
*V. cholerae*, Mouse intestinal loop	ZnO QDs	-	Coadministered kanamycin	[90]
*S. epidermidis*, *S. aureus*, *K. pneumonia*, *E. coli*	ZnO (spheres, plates, pyramids)	-	-	[91]
MRSA	Nano- and micro-sized ZnO coatings	-	Coadministered, Gent, Trim, Rif, Cip, Van	[92]
*S. aureus*, *E. coli*, *mice*	NHS, ZnO NRs, ZnO NSs	-	-	[93]

^a^ EPEC: Enteropathogenic *Escherichia coli*; *C. jejuni*: *Campylobacter jejuni*; *V. cholerae*: *Vibrio cholerae*; MRSA: Methicillin resistant *Staphylococcus aureus*; THP-1: Human monocytic cell; *M. tuberculosis*: *Mycobacterium tuberculosis*; *A. baumannii*: *Acinetobacter baumannii*; *S. aureus: Staphylococcus aureus*; *S. epidermidis*: *Staphylococcus epidermidis*; *K. pneumonia: Klebsiella pneumonia*; MSCL 302: *Enterococcus faecalis*; ATC_L3_C 7644: *Listeria monocytogenes*; O157:H7: *E. coli; P. aeruginosa*: *Pseudomonas aeruginosa*. ^b^ ZnO QDs: Zinc oxide quantum dots; Ag QDs: Silver quantum dots; Cu-ZnO NAs: Cu substituted ZnO nanoassemblies; ZnO-NPYs: ZnO nanopyramids with hexagonal base; ZnO QDs CVZnO: Crystal violet and zinc oxide nanoparticles; MPA@ZnO-PEP: Silica stabilized ZnO quantum dots with an antibacterial peptide fragment (UBI_29–41_) and a near infrared dye MPA (derived from hydrophilic indocyanine green); NHS: ZnO nanorods−nanoslices hierarchical structure; NRs: Nanorods; NSs: Nanoslices. ^c^ UV: Ultra violet; ^d^ Cip: Ciprofloxacin; Cef: Ceftazidime; Met: Methicillin; Van: Vancomycin; Gent: gentamicin; Trim: trimethoprim; and Rif: rifampicin.

**Table 3:** ZnO nanoplatforms for antifungal treatment.

Type of Fungi Used ^a^	Type of Device ^b^	Responsive Phenomena ^c^	Reference
*B. cinerea*, *P. expansum*	ZnO QDs	-	[97]
*A. saloni*, *S. rolfii*	ZnO QDs	-	[98]
*R. stolonifera*, *A. flavus, A*. *nidulans, T. harzianum*	ZnO QDs	-	[99]
*E. salmonicolor*	ZnO QDs	-	[100]
*A. niger*, *P. oxalicum*, *Paraconiothyrium* sp., *P. maculans*	Zn/Mg Oxide QDs	UV light	[101]
*A. fumigatus*, *C. albicans*	ZnO QDs	-	[102]
*C. albicans*	CS-LiA ZnO QDs	-	[105]
*R. stolonifera*, *P. expansum*	ZnO QDs	-	[106]
*C. krusei*	ZnO QDs	-	[107]

^a^*B. cinerea*: *Botrytis cinerea*; *P. expansum*: *Penicillium expansum*; *A. saloni*: *Alternaria saloni*; *S. rolfii*: *Sclerotium rolfsii*; *A. flavus*: *Aspergillus flavus*; *A. nidulans*: *Aspergillus nidulans*; *T. harzianum*: *Trichoderma harzianum*; *E. salmonicolor*: *Erythricium salmonicolor*; *A. niger*: *Aspergillus niger*; *P. oxalicum*: *Penicillium oxalicum*; *P. maculans*: *Pestalotiopsis maculans*; *A. fumigatus*: *Aspergillus fumigatus*; *C. albicans*: *Candida albicans*; *C. krusei*: *Candida krusei*. ^b^ ZnO QDs: Zinc oxide quantum dots; CS-LiA ZnO QDs: ZnO QDs coated by chitosan (CS) and functionalized with linoleic acid (LiA). ^c^ UV: Ultra violet.

**Table 4:** ZnO nanoplatforms for diabetes treatment.

Model Used	Type of Device ^a^	Drug/Antibiotic ^b^	Reference
Rats	ZnO QDs	-	[111]
Rats	ZnO QDs, ZnSO_4_	-	[112]
Rats	ZnO QDs	-	[113]
Rats	ZnO, CeO_2_, Ag QDs, MC	-	[114]
Rats	ZnO QDs	Coadministered Vildagliptin	[115]
Murine Pancreatic and Small Intestinal Extracts	ZnO QDs	Conjugated RSW	[116]

^a^ ZnO QDs: Zinc oxide quantum dots; CeO_2_ QDs: cerium oxide quantum dots; Ag QDs: silver quantum dots; MC: *Momordica charantia*. ^b^ RSW: Red Sandalwood.

**Table 5:** ZnO nanoplatforms with anti-inflammatory properties.

Model Used ^a^	Type of Device ^b^	Reference
AD model mouse	nZnO, bZnO	[118]
RAW 264.7	ZnO QDs	[119]
mice	ZnO QDs	[120]

^a^ AD: Atopic dermatitis; RAW 264.7: Murine macrophage cells. ^b^ nZnO: nano-sized ZnO; bZnO: bulk-sized ZnO; ZnO QDs: Zinc oxide quantum dots.

**Table 6:** ZnO nanoplatforms for wound healing.

Model Used ^a^	Type of Device ^b^	Reference
nHDF cells, SD rats	CZBs	[125]
PBMC, sheep fibroblast cells	SAGA-ZnO QDs hydrogels	[126]
HaCaT	CHT/SS/ZnO QDs, CHT/SS/LA	[128]
rats	CHT/gel/C4S/ZnO films	[129]

^a^ nHDF: Normal human dermal fibroblasts; SD: Sprague-Dawley; PBMC: Peripheral blood mononuclear cells; HaCaT: Aneuploid immortal keratinocyte cell. ^b^ CZBs: Microporous chitosan hydrogel/nano zinc oxide composite bandages; SAGA-ZnO QDs hydrogels; CHT/SS/ZnO QDs: Chitosan/silk sericin scaffolds combined with ZnO QDs; CHT/SS/LA: Chitosan/silk sericin scaffolds combined with lauric acid; CHT/gel/C4S/ZnO films: chitosan-based films containing gelatin, chondroitin 4-Sulphate and ZnO, respectively.
